# Micronutrient homeostasis in plants for more sustainable agriculture and healthier human nutrition

**DOI:** 10.1093/jxb/erac014

**Published:** 2022-02-04

**Authors:** Ana G L Assunção, Ismail Cakmak, Stephan Clemens, Manuel González-Guerrero, Adam Nawrocki, Sébastien Thomine

**Affiliations:** Department of Plant and Environmental Sciences, University of Copenhagen, 1871 Frederiksberg, Denmark; CIBIO-InBIO, Research Centre in Biodiversity and Genetic Resources, University of Porto, 4485-661 Vairão, Portugal; Faculty of Engineering and Natural Sciences, Sabanci University, 34956 Istanbul, Turkey; Department of Plant Physiology and Faculty of Life Sciences: Food, Nutrition and Health, University of Bayreuth, 95440 Bayreuth, Germany; Centro de Biotecnología y Genómica de Plantas (UPM-INIA), Universidad Politécnica de Madrid, 28223 Pozuelo de Alarcón (Madrid), Spain; PPC ADOB, 61-070 Poznań, Poland; Institute for Integrative Biology of the Cell (I2BC), Université Paris-Saclay, CEA, CNRS, 91198 Gif-sur-Yvette, France; Université Libre de Bruxelles, Belgium

**Keywords:** Essential metals, iron, micronutrients, nitrogen fixation, nutrition, photosynthesis, sustainability, zinc

## Abstract

The provision of sustainable, sufficient, and nutritious food to the growing population is a major challenge for agriculture and the plant research community. In this respect, the mineral micronutrient content of food crops deserves particular attention. Micronutrient deficiencies in cultivated soils and plants are a global problem that adversely affects crop production and plant nutritional value, as well as human health and well-being. In this review, we call for awareness of the importance and relevance of micronutrients in crop production and quality. We stress the need for better micronutrient nutrition in human populations, not only in developing but also in developed nations, and describe strategies to identify and characterize new varieties with high micronutrient content. Furthermore, we explain how adequate nutrition of plants with micronutrients impacts metabolic functions and the capacity of plants to express tolerance mechanisms against abiotic and biotic constraints. Finally, we provide a brief overview and a critical discussion on current knowledge, future challenges, and specific technological needs for research on plant micronutrient homeostasis. Research in this area is expected to foster the sustainable development of nutritious and healthy food crops for human consumption.

## Teaser

This review calls attention to the importance of plant micronutrient homeostasis for healthy nutrition of the growing human population, and for productive and sustainable agriculture, under increasing environmental constraints.

## Introduction

Plants need a range of essential micronutrients, including boron (B), chlorine (Cl), copper (Cu), iron (Fe), manganese (Mn), molybdenum (Mo), nickel (Ni), and zinc (Zn), for optimal growth (Marschner, 2012). Animals, including humans, also require micronutrients to be in good health and for their well-being (Suttle, 2010; Marschner, 2012). Micronutrient deficiencies are a widespread and growing problem in both crop plants and human populations worldwide. Mild or hidden micronutrient deficiencies likely limit crop yields in much wider areas than those where obvious symptoms occur. Insufficient amounts and low bioavailability of micronutrients in plant-based diets represent a major reason for the high prevalence of micronutrient deficiencies in human populations. Generally, there is a geographical overlap between micronutrient deficiencies in cultivated soils and those in human populations (Welch *et al.*, 2014). This indicates a close connection between the micronutrient status of plants and the health of the population. However, compared with other plant mineral nutrients, there is a general lack of concern about micronutrients with respect to both yield sustainability and the nutritional quality of food crops.

The purpose of this review is to raise awareness about the importance of micronutrient supply to plants, not only to improve yield and correct micronutrient deficiencies in humans, but also to improve agricultural sustainability. In this review, using Europe as an example, we first focus on the problem of micronutrient deficiencies in human populations, and the improvement or diversification of food crops needed to alleviate this public health issue. Then, we highlight how knowledge on plant micronutrient homeostasis could be used to improve crop yield and sustainability. Finally, we provide an overview of the knowledge on the molecular and physiological mechanisms of micronutrient homeostasis in plants, focusing mostly on Fe and Zn, and of the specific challenges in this area to address the issues of human nutrition and agricultural sustainability.

## Improving plant micronutrient content for human health

### Micronutrient deficiencies: not only a problem of developing countries

At the population level, micronutrient deficiencies are a major threat to human health. About one-third of the human population is globally affected by micronutrient deficiencies (also known as ‘hidden hunger’) that result most commonly from low dietary intake, mainly in developing regions where populations rely on plant-based diets from micronutrient-poor cereal crops (Harding *et al.*, 2018; Van Der Straeten *et al.*, 2020). Low dietary intake of micronutrients is also widely reported in European populations, especially among elderly people and children, with significant adverse impacts on health. Micronutrients such as Zn, Fe, selenium (Se), and iodine (I) are required for several critical functions, including cognition, development, the immune response, thyroid function, maintaining antioxidant activity, and the mitigation of chronic diseases (Bailey *et al.*, 2015; Steinbrenner *et al.*, 2015; Read *et al.*, 2019; Kieliszek *et al.*, 2021).

Aging is an unavoidable biological process that is often associated with a decline in physical and mental health. However, age-related impairments in well-being can be greatly alleviated by dietary adjustments (Ruxton *et al.*, 2016). According to European dietary survey studies, the diets commonly consumed by elderly people in Europe are rich in saturated fat, but very low in mineral elements such as magnesium (Mg), Zn, and Cu (Ruxton *et al.*, 2016). A systematic compilation of several databases containing data on over 7200 older adults (≥60 years of age) in 13 Western countries [including 10 countries in the European Union (EU)] showed that micronutrient deficiencies are a critical nutritional problem among older adults. Among the eight micronutrients investigated, Zn, Se, Cu, and I deficiencies were prevalent in elderly people. Notably, 31% of women and 49% of men studied were found to consume less dietary Zn than the recommended intake (Vural *et al.*, 2020).

Children are highly vulnerable to micronutrient deficiencies (Van Der Straeten *et al.*, 2020; Vassilakou, 2021). For example, Fe deficiency was found to be prevalent among 12- to 36-month-old children in Germany, the Netherlands, and the UK (Akkermans *et al.*, 2016). Similarly, Fe deficiency was reported in 18-month-old children in Denmark and in children aged 1–11 years in Spain (Andersen *et al.*, 2019; López-Ruzafa *et al.*, 2021). Based on an extensive literature review, Eussen *et al* (2015) evaluated the relevance of Fe deficiency in European children aged 6–36 months, and showed that it ranged between 3% and 48%, with children living in eastern Europe having a higher prevalence of Fe deficiency than those living in western Europe (Eussen *et al.*, 2015). However, this nutritional problem usually receives little attention or is often overlooked in Europe.

Additionally, the increase in the popularity of vegetarian and vegan diets worldwide will impact nutritional outcomes, especially in terms of micronutrients (Schüpbach *et al.*, 2017; Sebastiani *et al.*, 2019; Bakaloudi *et al.*, 2021). It is estimated that ~5% of the population of Europe and 19% in Asia (Hargreaves *et al.*, 2021) excludes animal products containing a high amount of bioavailable micronutrients in favor of plant products that are rich in antinutrient compounds (e.g. phytate), which reduce micronutrient bioavailability in the human body (Gibson *et al.*, 2018). In addition, the high alcohol consumption that is common in Europe significantly interferes with the intestinal absorption of micronutrients and contributes to Zn, Fe, and Se deficiencies in human populations (Barve *et al.*, 2017; Hillesund *et al.*, 2021).

### Towards crops with higher micronutrient content and bioavailability for food and feed

Several strategies can be proposed to achieve better micronutrient delivery through plant-based products: (i) major crops can be bred for higher micronutrient content and availability (Sayre *et al.*, 2011; Ludwig and Slamet-Loedin, 2019); (ii) orphan or indigenous crop species or macroalgae can be screened for micronutrient content and availability, and introduced into the European diet (García-Casal *et al.*, 2007; Masuda *et al.*, 2015); (iii) plant species with outstanding micronutrient content, such as Zn-hyperaccumulating species, can be cultivated and used as an additive in other foods (Clemens, 2017). The first strategy would be most convenient from an agronomical and social point of view because it is based on crops that are already widely used, but so far this approach has resulted in only small improvements (Connorton and Balk, 2019; Kawakami and Bhullar, 2021). Nonetheless, knowledge is advancing on the regulatory networks of micronutrient homeostasis in plants, which could lead to the discovery of molecular switches that could have a significant impact on micronutrient accumulation. Once such molecular switches have been identified, existing genetic resources (e.g. germplasm collections, natural reserves of biodiversity) may be more efficiently screened for genetic variation that optimizes micronutrient use efficiency and accumulation. The second and third strategies, although probably very efficient, would require the domestication of novel species and changes of dietary habits in the population.

In working towards improving micronutrient bioavailability in the edible parts of crop plants, new challenges for research on micronutrient homeostasis in plants must be met. In particular, knowledge on the mechanisms of micronutrient distribution within the plant, the metal specificity of ligands and transporters, and metal speciation, especially in edible organs, is needed, as detailed below. Moreover, understanding how the chemical forms of micronutrients are altered by processing, such as sprouting, milling, and cooking, and how these changes affect their uptake by the digestive system is essential. To date, there is limited information about these aspects, and further research needs to be performed in close collaboration with nutrition scientists and physicians to determine accurately how crop varieties with improved micronutrient content and availability impact human health (Cakmak *et al.*, 2020).

## Improving plant micronutrient status for yield and sustainability

### Soil micronutrient deficiency: heterogeneous and not sufficiently documented

Soil micronutrient deficiencies significantly affect crop yield and quality for human consumption in many areas in Europe, with sharp regional differences (Sinclair and Edwards, 2008; Alloway, 2009). For example, Mn deficiency in soils and plants has been reported in the UK and the northern part of Europe (Hebbern *et al.*, 2005; Alejandro *et al.*, 2020), while in south European and Mediterranean countries, Zn and Fe deficiencies are common, although often without causing visible symptoms in plants (Abadía *et al.*, 2004; Rashid and Ryan, 2004; Moreno-Lora and Delgado, 2020). Soil micronutrient deficiency is also widespread in dryland regions of Europe. In a survey study conducted in six European countries, Portugal, Spain, Italy, Hungary, Greece, and Malta, Moreno-Jiménez *et al.* (2021) showed that 60% and 28% of the investigated soils contained lower than adequate levels of Fe and Zn, respectively. Given that an important fraction of crop production takes place in dryland regions, improving the Fe and Zn nutritional status of crop plants growing in these regions is of great importance. Soil deficiency of phytoavailable Zn and Fe results in important impairments not only in crop productivity but also in crop nutritional quality.

To promote awareness about regional problems of micronutrient deficiencies and facilitate their targeted alleviation, it would be important to map, at a fine scale, the phytoavailable concentrations of mineral micronutrients in soils to identify areas where they limit crop production. Such knowledge would also allow precision agriculture with micronutrient fertilization treatments tailored to the particular conditions. In the context of agroecology, efficient intercropping systems, such as maize and bean, used for centuries in Central America, could be scientifically designed (Lopez-Ridaura *et al.*, 2021). Alternatively, micronutrient-rich green fertilizers could be applied to limit the use of synthetic fertilizers and pesticides. In contrast, soil-free urban or greenhouse intensive agriculture requires the fine tuning of plant micronutrient status through the use of fully controlled nutrient solutions (Eldridge *et al.*, 2020).

The fine mapping of micronutrient availability could be achieved through several complementary means: the mining of soil-related databases; the use of indicator plants, possibly through participative projects; and the design of devices to evaluate *in situ* crop micronutrient status using portable X-ray fluorescence equipment, or remotely, using drone-based hyperspectral analyses (Dobbels and Lorenz, 2019; Qu *et al.*, 2022). The European Soil Data Centre (ESDAC) is the center for soil-related data in Europe and runs a project responsible for a harmonized and regular survey of top soils across all EU member states, named the Land Use/Cover Area frame statistical Survey (LUCAS). This survey represents an attempt to build a consistent spatial database of the soil cover across the EU, based on standard sampling and analytical procedures. In addition to soil properties, the macronutrients, including phosphorus (P), nitrogen (N), and potassium (K), are analyzed. A report on a Cu survey has recently been published within the LUCAS project (Ballabio *et al.*, 2018). In the future, the LUCAS survey should also incorporate the analysis of phytoavailable concentrations of micronutrients such as Fe, Zn, B, Mn, and Se.

### Correcting micronutrient deficiencies with fertilizers

In the past decades, key knowledge has been produced on the formulation of fertilizers to supply micronutrients to plants. Furthermore, innovative strategies have emerged, such as seed priming or coating with micronutrients (Farooq *et al.*, 2012). Synthetic chelators that bind Fe or Zn with high affinity while making these metals available for uptake by plants, such as HBED [*N*,*N*ʹ-di(2-hydroxybenzyl)ethylenediamine-*N*,*N*ʹ-diacetic acid monohydrochloride], are now used in the field (Abadía *et al.*, 2011). The agronomic effectiveness of the chelated micronutrients is much higher than that of the inorganic forms of the corresponding micronutrients when applied on calcareous soils (Gangloff *et al.*, 2002). At the global scale, only 6% of micronutrient fertilizers are applied as chelates, mostly in Europe, west Asia, and North America. Correction of micronutrient deficiencies in plants by using chelates is an expensive practice. According to Abadia *et al*. (2004), the cost of correcting Fe deficiency in plants grown in Mediterranean countries represents an important economic burden on farmers of about €80 million to €100 million per year (Abadía *et al.*, 2004). Therefore, the optimization of the mode of application of the chelated micronutrients is an important topic for studies. In this regard, foliar spray has many advantages as it minimizes the quantities applied, increases efficacy, and prevents loss of micronutrients by precipitation in soils. Foliar application of Zn is also more effective than soil application in increasing Zn concentrations in grains (Cakmak *et al.*, 2010a). Moreover, foliar spray can be applied locally as a precise treatment and in combination with other plant-protection products. Studies on the mechanisms of chelated metal uptake by the roots and the leaves may allow improvement of their efficiency and limit the costs to farmers.

### Harnessing micronutrient homeostasis for sustainable yield and protein production

**Fig. 1.**
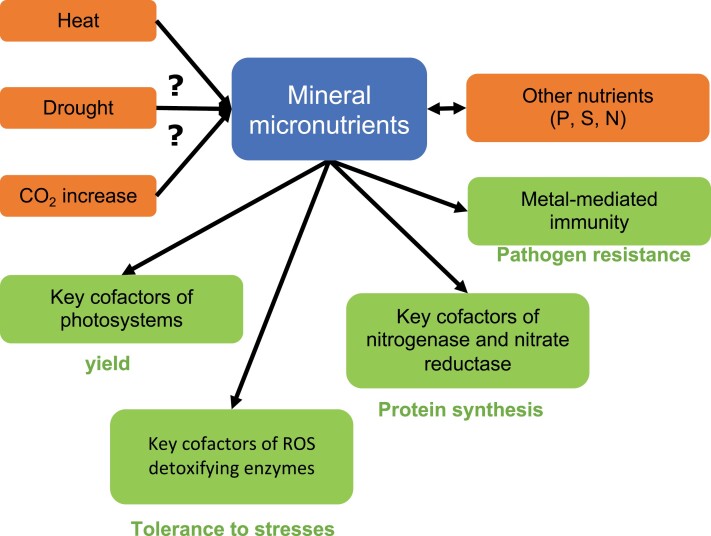
Micronutrient interactions in the face of global change. How micronutrition is influenced by heat, drought, and atmospheric CO_2_ concentration, all of which are expected to increase during climate change, needs to be investigated. The mechanisms underlying the interactions of micronutrients with each other and with macronutrients need to be deciphered. In turn, micronutrients act as key cofactors in processes that help mitigate the impact of global change, such as photosynthesis, nitrogen assimilation, reactive oxygen species detoxification, and immunity.

Global change calls for more sustainable and more productive agriculture, in which less N fertilizer is used and crops are more resilient to environmental constraints. Optimal micronutrient uptake and distribution are essential to achieve this goal, as photosynthesis, N nutrition, and resilience to abiotic and biotic stresses largely rely on sufficient mineral micronutrient supply (Fig. 1). For this to become a reality, it is essential to advance knowledge on the molecular and cellular pathways of micronutrients, from their acquisition from soils to their incorporation as cofactors in metalloproteins, and their regulation.

Mineral micronutrients are key to maintaining optimal photosynthesis and therefore crop productivity. Accordingly, many mutants identified in screens for defective photosynthesis are affected in genes encoding metal transport components (Schneider *et al.*, 2016; Eisenhut *et al.*, 2018; Zhang *et al.*, 2018; Schmidt *et al.*, 2020). The photosynthetic electron transport chain requires high levels of Fe, Mn, and Cu (Schmidt *et al.*, 2020). Photosystems are quantitatively among the most abundant enzymatic systems in ecosystems. Chloroplasts contain ~80% of total cellular Fe in a mesophyll cell. Up to 50% of the plant Cu is found in chloroplasts, bound mainly to plastocyanin, which is an essential electron carrier in the photosynthetic system (Schmidt *et al.*, 2020). To improve the supply of micronutrients to the photosystems, the mechanisms of their uptake into leaf cells and transport through the chloroplast envelope and thylakoid membranes need to be deciphered.

Key enzymes for N fixation and assimilation rely on Fe and Mo for their activity: nitrogenase, which is required for atmospheric N_2_ fixation, and nitrate reductase, which catalyzes the first step in the assimilation of N into proteins, both require Fe- and Mo-containing cofactors. Accordingly, Fe deficiency specifically decreases nitrate reductase activity with no change in glutamine synthetase activity (Borlotti *et al.*, 2012) and affects nodule initiation and development (Brear *et al.*, 2013). Owing to their high content of nitrogenase, which requires 38 atoms of Fe, and leghemoglobin, a heme protein, nodules represent a major sink accounting for ~35% of the total Fe in a legume (Burton *et al.*, 1998; Brear *et al.*, 2013; Burén *et al.*, 2020). To improve N fixation, the mechanisms responsible for the distribution of the metal cofactors of enzymes involved in N fixation and assimilation need to be determined.

To maintain a sustainable agricultural yield, it is also essential to harness the functions of micronutrients in tolerance to biotic and abiotic constraints. This includes understanding the roles of micronutrients in plant resilience to abiotic stresses, through their antioxidant role, for example, and understanding the competition for micronutrients between the host and invading pathogens and how it is regulated or manipulated during the plant immune response. With global change, crops are expected to be increasingly affected by abiotic stresses such as heat, drought, high light, and radiation. Whether metal micronutrients, which are key players in redox reactions, can mitigate or aggravate the effect of global change, and how nutrition with Zn, Mn, Fe, and B could mitigate oxidative stress, needs to be explored (Fig. 1). On the other hand, the atmospheric CO_2_ concentration is increasing steadily, and tentative evidence indicates that this is causing a decrease in the micronutrient nutritional value of plant products (Dong *et al.*, 2018). Knowledge on micronutrient homeostasis may also be useful to fight plant diseases. There is growing evidence that micronutrients are involved in plant immunity against pathogens (Aznar *et al.*, 2015; Verbon *et al.*, 2017; Cesco *et al.*, 2020; Escudero *et al.*, 2022; Morina *et al.*, 2021). In addition, micronutrients, especially B, Cu, and Mn, are important for the structural stability and mechanical resistance of the cell wall, which is the first barrier against pathogens (Krzesłowska, 2011; Malinovsky *et al.*, 2014). However, the underlying mechanisms need to be better understood to allow the inclusion of micronutrient nutritional supply as a support for crop protection, which may open a new venue of business for micronutrient fertilizer companies. Furthermore, better knowledge of the roles of micronutrients in defense responses may allow mitigation of the overuse of exogenous Cu to protect plants against fungal infection that has led to persistent pollution in vineyards, for example (Provenzano *et al.*, 2010).

## Overview of current knowledge and future challenges for research on plant mineral micronutrient homeostasis

Plants need micronutrients for specific biochemical processes such as photosynthesis or N assimilation, in addition to more general processes such as protein synthesis and function, DNA replication, respiration, or reactive oxygen species detoxification (Marschner, 2012). To ensure a sufficient micronutrient supply in plants, a highly specialized and regulated network is in place to optimize uptake from soil, distribution throughout the plant, delivery to sink organs, and allocation to specific metalloproteins. This is particularly challenging considering the prevalent metal micronutrient deficiencies in soils described above, and the toxic effects of concentrations slightly above physiological levels.

So far, research on the molecular mechanisms of plant micronutrient acquisition and use efficiency has been strongly focused on the two main plant models, *Arabidopsis thaliana* and rice. In the future, information from these models needs to be extended to other important crops.

### Micronutrient uptake

For most micronutrients, the molecular mechanisms of uptake by plants have been elucidated in the past decades (Olsen and Palmgren, 2014; Connorton *et al.*, 2017; Alejandro *et al.*, 2020; Stanton *et al.*, 2021). The main metal micronutrient transporters have been identified, as has the role of rhizosphere acidification in metal acquisition. In the past few years, the importance of root exudates in the mobilization of essential micronutrients in dicots has been uncovered. While it was already known that cereals acquire Fe by secreting phytosiderophores into the rhizosphere, the central role of secreted coumarins in the mobilization of Fe in the vicinity of growing roots has been highlighted in several dicot species (Sisó-Terraza *et al.*, 2016; Tsai and Schmidt, 2017), with possible implications for the acquisition of other micronutrients, as well as shaping the bacterial communities associated with the roots (Stringlis *et al.*, 2018). In addition, recent studies have reported the existence of specific mechanisms for metal uptake from arbuscular mycorrhizal fungi, which are of particular relevance in natural ecosystems and organic agrosystems (Senovilla *et al.*, 2020). Root mycorrhizal colonization contributes up to 50% of plant Zn uptake (Watts-Williams *et al.*, 2015). Moreover, decreasing root mycorrhizal colonization results in massive increases in cadmium (Cd) accumulation in wheat (Yazici *et al.*, 2021). Particular attention should thus be given to the abundance and activity of mycorrhizal fungi in agricultural soils, to manage micronutrient nutrition and limit Cd accumulation in food crops (Ma *et al.*, 2021).

### Micronutrient distribution within the plant

Within the plant, micronutrients are delivered through the sap to sink organs, typically the youngest leaves and seeds. This is done by transporters moving these nutrients across membranes, and by small organic ligands that maintain their solubility in solution and prevent mis-metallation (Olsen and Palmgren, 2014; Connorton *et al.*, 2017). Whereas many genes involved in micronutrient mobilization and uptake from the soil have been identified, for most micronutrients, the mechanisms for loading into the xylem and the phloem, as well as the ligands in conducting tissues, are still mostly unknown. The transporters localized in the nodes and tiller buds play fundamental roles in the distribution of micronutrients within plants, especially in the delivery of micronutrients to the younger leaves and seeds, and should be targeted for more research (Durbak *et al.*, 2014; Shao *et al.*, 2018; Mu *et al.*, 2021). The mechanisms of micronutrient distribution to the edible organs are still only partially understood (Andresen *et al.*, 2018). Improving the micronutrient content in the edible parts of crop plants will require better knowledge of the mechanisms of micronutrient distribution to the organs and tissues in order to be able to breed or engineer plants for targeted micronutrient allocation.

Moreover, the process of metal recycling and reuse in the transition from vegetative to reproductive growth remains largely unaddressed. The study of metal remobilization from senescing leaves appears to be a promising area to modulate metal accumulation in seeds (Mari *et al.*, 2020). Modulating leaf senescence and nutrient recycling by autophagy in wheat or Arabidopsis leaves leads to change in the contents of both protein and mineral micronutrients in seeds (Uauy *et al.*, 2006; Chen *et al.*, 2019; Pottier *et al.*, 2019).

Another critical process that calls for further investigation is the mechanism of mineral micronutrient loading into seeds. At the moment, only fragmentary information on micronutrient entry into seeds is available. Metal-pumping ATPases have been shown to play an important role in releasing Zn from the mother tissues before its entry into the seed (Olsen *et al.*, 2016). The secretion of ascorbate is used to reduce Fe and allow its uptake by the embryo (Grillet *et al.*, 2014). Developing this research will require detailed characterization of the dynamics of micronutrient distribution in plant organs and tissues using elemental imaging approaches as well as metal isotopes as tracers (Sheraz *et al.*, 2021).

### Micronutrient supply to endosymbionts

Plant endosymbionts, particularly those associated with N fixation, are major metal sinks for which specific metal-delivery systems have evolved (Tejada-Jiménez *et al.*, 2017; Brear *et al.*, 2020; Escudero *et al.*, 2020). To improve N nutrition through endosymbiotic bacteria, the genes involved in transport pathways that supply micronutrients to the nitrogenase of symbiotic bacteria, enclosed in symbiosomes in the nodules of legume species, or of endophytic bacteria that colonize the extracellular space in other species, including wheat and other cereals, need to be identified. Moreover, the mechanisms controlling micronutrient allocation to two opposite sinks (leaves and nodules) remain largely unknown, with important physiological implications on how legumes dynamically distribute essential limiting cofactors for photosynthesis and for N fixation.

### Micronutrient interactions

Besides their importance for N metabolism, micronutrients also strongly interact with each other and with several macronutrients. In fact, deficiency symptoms are often related to nutritional imbalances rather than a lack of a single micronutrient per se. There are important interactions between micronutrient homeostasis and other mineral nutrients, such as inorganic phosphate (Pi) with Zn and Fe (Briat *et al.*, 2015; Dong *et al.*, 2017; Hanikenne *et al.*, 2021). High Pi has an inhibitory effect on root Zn uptake (Watts-Williams *et al.*, 2015; Ova *et al.*, 2015). On the other hand, Zn deficiency increases Pi uptake (Kisko *et al.*, 2018). Zn and Fe root uptake, as well as their root-to-shoot transport and remobilization, are positively affected by N fertilization (Kutman *et al.*, 2011). In line with this, the concentrations and localizations of Zn, Fe, and proteins in seeds are closely correlated (Cakmak *et al.*, 2010b). In contrast, Mo uptake is severely affected by sulfur (S) fertilization, resulting in Mo deficiency (Shinmachi *et al.*, 2010; Maillard et al., 2016*a*, *b*). To determine the molecular bases of these interactions, the metalloproteins involved and their biological roles need to be elucidated.

A major risk encountered in biofortification strategies targeting mineral micronutrients is enhancement of the accumulation of chemically similar toxic trace elements (Kawakami and Bhullar, 2021). In rice, enhancing the expression of a transporter involved in Cd sequestration in root vacuoles, or knocking out a gene involved in Cd uptake in roots, have proved to be efficient strategies to prevent Cd accumulation in grains (Ueno *et al.*, 2010; Ishikawa *et al.*, 2012). Because of their chemical similarities, micronutrient metals compete with non-essential metals for transport and binding to ligands. Addressing these fundamental scientific questions requires in-depth biochemical knowledge of the substrate specificities and relative affinities of proteins and transporters for micronutrients and toxic elements with similar chemical properties (e.g. Zn and Cd). The determinants for metal specificity, particularly among divalent metals, need to be better defined to promote the exclusion of non-biogenic metals (Pottier *et al.*, 2015).

### Micronutrient speciation

The major metal ligands in plants, such as citrate or nicotianamine, and their role in micronutrient transport between cells and organs have been established (Clemens, 2019). Enhanced accumulation of the metal-binding molecule nicotianamine through the activation of the gene encoding nicotianamine synthase allowed an increase in the content of bioavailable Fe and Zn in rice grains (Lee et al., 2009, 2011). Nevertheless, improving micronutrient content in the edible parts of crop plants and optimizing metal distribution for photosynthesis and N metabolism requires a better knowledge of the mechanisms of micronutrient speciation and subcellular distribution. The analysis of micronutrient speciation, including the determination of the metalloproteome and metallome for each essential metal, using analytic and spectroscopic methods is crucial to achieve this goal (Flis *et al.*, 2016). The control of metal speciation requires knowledge on the mechanisms that drive micronutrient allocation to distinct cell compartments, as speciation varies among the different cellular compartments. For example, Fe is often stored as an insoluble complex with phytate in the vacuole, whereas it is stored as bioavailable ferritin in the plastids (Mari *et al.*, 2020).

### Regulation of metal homeostasis

Progress has also been made in understanding the regulation of micronutrient acquisition: transcription factors (TFs) controlling plant responses to Fe, Zn, and Cu deficiency have been identified (Assunção *et al.*, 2010; Bernal *et al.*, 2012; Kobayashi and Nishizawa, 2012). These findings highlight the importance of the transcriptional control of genes involved in micronutrient transport and distribution in the plant response to micronutrient deficiencies. In addition, more information is emerging on the mechanisms through which plants perceive micronutrient status, and on micronutrient status signaling at the systemic plant level, as has recently been reported for Zn and Fe (Dubeaux *et al.*, 2018; Grillet *et al.*, 2018; Sinclair *et al.*, 2018; Lilay *et al.*, 2021). In the case of Fe, a complex network involving over 10 bHLH TFs has been uncovered (Gao and Dubos, 2021). Interestingly, the stability of several of these TFs is controlled by Fe-dependent E3 ligases (Rodríguez-Celma *et al.*, 2019). A recent publication indicates that phosphorylation of one of these bHLH TFs is responsible for recognition by E3 ligases, raising the possibility that some kinases mediate the Fe response (Kim *et al.*, 2019). For Zn, F-group bZIP TFs have been identified as the central regulators of the deficiency response. Under Zn deficiency, they transcriptionally activate Zn transporters and ligand-producing enzymes, which increase the plant’s Zn uptake and distribution capacity as an adaptation to low Zn availability (Assunção *et al.*, 2010). The F-bZIP TF itself acts as a sensor of cellular Zn status by directly binding to Zn ions (Lilay *et al.*, 2021). In the case of Cu, the Arabidopsis SPL7 TF plays a pivotal role in the response to Cu deficiency through the transcriptional activation of genes involved in Cu uptake and mobilization. The TF also mediates miRNA-dependent down-regulation of Cu metalloprotein transcripts, as a mechanism to spare Cu under conditions of deficiency (Yamasaki *et al.*, 2009; Bernal *et al.*, 2012; Garcia-Molina *et al.*, 2014).

Advancing knowledge on the regulatory networks that control micronutrient acquisition at the cellular and plant levels will help to improve micronutrient use efficiency and micronutrient content in crops. Moreover, the long-distance signaling mechanisms that allow adjustment of the uptake and redistribution of micronutrients according to the need of the sink organs should also be elucidated. This is important to optimize micronutrient use efficiency and also to improve plant responses to micronutrient fertilizers.

### Specific approaches required to decipher mineral micronutrient homeostasis

All these breakthroughs have been facilitated by advances in micronutrient visualization and quantification. In the past decades, ionomic screens based on atomic absorption and emission spectroscopies as well as mass spectrometry have been used to quantify elements in large collections of plants at a tissue/organ level. These approaches have proved useful for identifying genes involved in micronutrient homeostasis in model species (Huang and Salt, 2016; Campos *et al.*, 2017; Yang *et al.*, 2018). Spatial resolution has been achieved using synchrotron X-ray fluorescence (sXRF) or micro-particle induced X-ray emission (micro-PIXE) to map micronutrient localization in the nanomolar to micromolar range. Improved synchrotron lines with cryo-facilities have become the gold standard in terms of sensitivity, resolution, and minimal preparation artefacts. When associated with X-ray absorption spectroscopy, synchrotron-based approaches also provide information about metal micronutrient speciation together with the localization data (Escudero *et al.*, 2020) and can be used to analyze the efficiency of micronutrient fertilization (Ajiboye *et al.*, 2015). Other imaging techniques based on mass spectrometry, such as secondary ion mass spectroscopy (SIMS), laser ablation inductively coupled plasma-mass spectrometry (LA-ICP-MS), or matrix-assisted laser desorption/ionization (MALDI) imaging, have been used to reveal micronutrient localization (Moore *et al.*, 2018; Detterbeck *et al.*, 2020). They provide isotopic information allowing dynamic analysis after isotopic labeling and, in some cases, allow *in situ* analysis of metal complexes. Finally, metal-sensitive fluorescent probes, such as Förster resonance energy transfer (FRET) sensors, dynamically monitor labile micronutrient pools *in vivo* (Lanquar *et al.*, 2014). In addition, analytical chemistry methods combining chromatography with mass spectrometry and elemental analysis, such as size-exclusion chromatography (SEC)-ICP-MS, have identified metal complexes with small molecules or with proteins (Cakmak *et al.*, 2010a; Flis *et al.*, 2016; Küpper *et al.*, 2019). Ultimately, the development of these approaches should allow the determination of the complete spectrum of micronutrient metal complexes with small molecules (metallome), and the full complement of metalloproteins (metalloproteome) for any given biological sample. This knowledge is essential to fully understand the role of micronutrients in living cells and to improve their bioavailability to ultimately address micronutrient deficiencies in human populations.

## Conclusion

Mineral micronutrient uptake and use efficiency in plants are still underutilized traits in crop breeding programs, even though they have the potential to improve both the sustainability of crop production and the quality of food and feed. To alleviate micronutrient deficiency problems in humans and in livestock, supplementation and fortification methods are most commonly recommended. However, these methods are unsustainable because they need continued financing, monitoring, and dedicated logistic services. Breeding food plants for higher mineral micronutrient contents appears to be a more sustainable solution to reduce the global prevalence of micronutrient deficiencies, which are expected to increase as the populations of developed countries move toward more plant-based diets. Moreover, breeding for mineral micronutrient use efficiency will also allow sustainable crop production, which is required to face global change. In this context, there is a need to encourage research and innovation in this field to ensure the well-being of human populations and the competitiveness of the agri-food industry. Understanding the molecular and physiological mechanisms of micronutrient homeostasis, including the regulation of the acquisition, transport, and distribution of micronutrients in food crops, is essential to identify sustainable solutions to micronutrient deficiencies in human populations.
